# Enzymatic browning: The role of substrates in polyphenol oxidase mediated browning

**DOI:** 10.1016/j.crfs.2023.100623

**Published:** 2023-10-20

**Authors:** Andrew Tilley, Mark P. McHenry, Julia Anwar McHenry, Vicky Solah, Kirsty Bayliss

**Affiliations:** a School of Medical, Molecular & Forensic Sciences, College of Environmental & Life Sciences, Murdoch, 6150, Western Australia, Australia; b Food Futures Institute, Murdoch University, 90 South Street, Murdoch, 6150, Western Australia, Australia; c Harry Butler Institute, Murdoch University, 90 South Street, Murdoch, 6150, Western Australia, Australia; d Mt Lindesay, 56 McHenry Lane, Scotsdale, 6333, Western Australia, Australia

**Keywords:** PPO, Browning, Oxidation, Phenolic, Polyphenol, Vegetables, Chlorogenic acid (pubchem CID: 1794427), Cysteine (pubchem CID: 5862), (−)-Catechin (pubchem CID: 73160), Ferulic acid (pubchem CID: 445858), Sinapic acid (pubchem CID: 637775), Catechol (pubchem CID: 289), Gallic acid (pubchem CID: 370)

## Abstract

Enzymatic browning is a biological process that can have significant consequences for fresh produce, such as quality reduction in fruit and vegetables. It is primarily initiated by polyphenol oxidase (PPO) (EC 1.14.18.1 and EC 1.10.3.1) which catalyses the oxidation of phenolic compounds. It is thought that subsequent non-enzymatic reactions result in these compounds polymerising into dark pigments called melanins. Most work to date has investigated the kinetics of PPO with anti-browning techniques focussed on inhibition of the enzyme. However, there is substantially less knowledge on how the subsequent non-enzymatic reactions contribute to enzymatic browning. This review considers the current knowledge and recent advances in non-enzymatic reactions occurring after phenolic oxidation, in particular the role of non-PPO substrates. Enzymatic browning reaction models are compared, and a generalised redox cycling mechanism is proposed. The review identifies future areas for mechanistic research which may inform the development of new anti-browning processes.

## Highlights

• Polyphenol oxidase (PPO) from plants primarily exhibit catechol oxidase activity.
• PPO activity in complex systems is discussed.
• Oxidative coupling of PPO products facilitates the formation of brown pigments.
• A generalised redox cycling mechanism for PPO product polymerisation is proposed.
• Regulating non-PPO substrates may offer novel solutions to minimise browning.

## Graphical abstract

**Figure:**
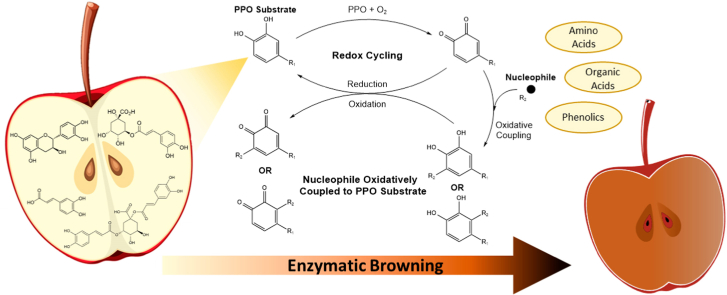

## 1 Introduction

Enzymatic browning of fresh produce is ubiquitous in the food supply chain, with both positive and negative consequences. While enzymatic browning is fundamental to the processing of products such as black tea, coffee, and cocoa, it is detrimental for fruit and vegetables (Debelo et al., 2020). Often caused by physical damage during handling it results not only in changes to colour, but also texture, flavour, and nutritional content (Lante et al., 2016). As a result, enzymatic browning is a significant contributor to food waste at all stages of the supply chain (Queiroz et al., 2008).

Polyphenol oxidase (PPO) is widely accepted to be the primary enzyme responsible for initiating the enzymatic browning process and so it has been the subject of several reviews. The biochemistry, cellular distribution, and biological function of PPO was extensively covered by Mayer (2006). In particular, the potential role of PPO in plant resistance to pathogens was discussed. While the oxidation of phenolic compounds was considered to be part of this defence mechanism, the resulting formation of melanin was not discussed. Rinaldo et al. (2010) reviewed the polyphenol composition of tropical and sub-tropical fruit, including their degradation due to the action of PPO. Of particular focus was the change to polyphenol content observed during postharvest handling and storage. However, how oxidation of the polyphenols by PPO resulted in enzymatic browning was not elucidated. A recent review by McLarin and Leung (2020) examined PPO substrate specificity and how this is regulated by the protein structure, but the fate of substrates after reaction with PPO was not within the scope of their review. Each of these reviews focussed on the biochemistry and catalytic mechanism of PPO, yet the products of PPO activity undergo further non-enzymatic reactions prior to forming brown pigments (Vissers et al., 2017).

These non-enzymatic reactions and the relative contribution of different substrates to the brown end products has, to our knowledge, not been reviewed. The purpose of this review was to evaluate the current knowledge and recent advances in understanding PPO-mediated browning. Particular focus was given to the non-enzymatic reactions occurring after oxidation of phenolics by PPO. Proposed reaction models were compared with a view to consolidating the current knowledge. Finally, the relative contributions of different substrates to the extent of enzymatic browning were reflected on.

## 2 Polyphenol oxidase and its substrates

### 2.1 Enzyme kinetics and mechanism of action

**Fig. 1:**
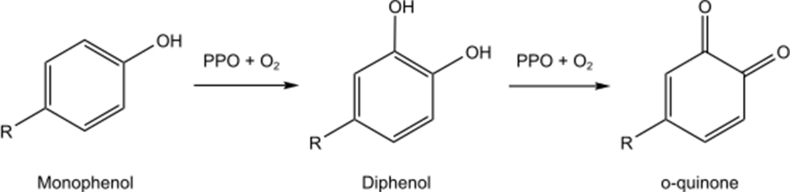
Hydroxylation and oxidation reactions catalysed by polyphenol oxidase.

Enzymatic browning is initiated by the type 3 copper protein PPO, that has been reported widely in bacteria, fungi, plants, and mammals (Li et al., 2019). Plant derived PPOs contain a highly conserved catalytic centre. This centre contains two copper ions, termed CuA and CuB, that are each coordinated by three histidine residues (CuA: H_A1-3_; CuB: H_B1-3_) (Molitor et al., 2015; Prexler et al., 2018). PPO can function as either a tyrosinase (EC 1.14.18.1) or catechol oxidase (EC 1.10.3.1). Tyrosinase can catalyse two different reaction cycles, the first being the *ortho*-hydroxylation of monophenols to diphenols with subsequent oxidation of the diphenol to an *o*-quinone; the second being the oxidation of an *o*-diphenol to an *o*-quinone. In contrast, catechol oxidase is only able to catalyse the oxidation of *o*-diphenols to *o*-quinones (Fig. 1.) (McLarin and Leung, 2020).

Many phenolic compounds possess an *o*-diphenolic group, making them potential substrates for PPO. Substrate specificity has been characterised for PPO derived from a wide range of fruit, vegetables and other plant products (Table 1.). Interestingly, the PPO derived from these foods has been reported to only exhibit catechol oxidase activity. Virador et al. (2010) made a similar observation, that cresolase (tyrosinase) activity is typically only observed in fungal and mammalian PPOs. However, the recent work by Li et al. (2019) and Derardja et al. (2022), using recombinant PPO from grape and apricot, suggests that plant derived PPOs can exhibit both activities, albeit with tyrosinase activity being substantially lower.

**Table 1:** Substrate specificity of polyphenol oxidase from different plant sources.

Source	Substrates	*V*_max_ (U mL^−1^ min)	*K*_*m*_ (mM)	*V*_max_/*K*_*m*_ (U min^−1^ mM^−1^)	*k*_*cat*_/*K*_*m*_ (s^−1^ mM^−1^)	Reference
**Fruits**						
Marula fruit (Sclerocarya birrea subsp. caffra)^a^	Catechol	122.0	4.99	24.2		Mdluli (2005)
	4-methylcatechol	69.5	1.45	47.9		
	3,4-dihydroxyphenylpropanoic acid (+)-catechin	75.1	3.73	20.1		
		54.2	1.41	38.5		
Jackfruit (*Artocarpus heterophyllus*)	Catechol	109.9	8.2	13.4		Tao et al. (2013)
	4-methylcatechol	82.1	18.2	4.5		
	Tyrosine	ND	ND	ND		
	Pyrogallol	ND	ND	ND		
	Gallic acid	ND	ND	ND		
Apricot fruit(*Prunus* armeniaca cv. Bulida)	Catechol	210	5.3		40	Derardja et al. (2017)
	Chlorogenic acid	1400	2.7		500	
	4-methylcatechol	700	2.0		340	
	Pyrogallol	590	11.0		50	
Apricot fruits and leaves (Prunus armeniaca, cv. Bafi)	Tyramine		4.48		0.318	Derardja et al. (2022)
	Dopamine		1.61		99.8	
	Catechol		3.29		48.4	
	L-3,4-dihydroxyphenylalanine Chlorogenic acid		7.16		17.5	
			1.52		141.2	
	Neochlorogenic acid		1.47		181.0	
	(+)-catechin		1.215		246.3	
	(−)-epicatechin		1.343		218.5	
	Procyanidin B2		13.3		7.79	
Kirmizi Kismis grape (*V. vinifera*)	4-methylcatechol	2000	4.8	416.66		Kaya and Bağci (2021)
	Catechol	1666.6	18.33	90.91		
	L-tyrosine	ND	ND	ND		
Blueberry (*Vaccinium corymbosum*)^b^	Catechol	187.90	6.55		182.72	Wei et al. (2022)
	Protocatechuic acid	ND	ND		ND	
	3,4-dihydroxyphenylacetic acid	28.75	15.44		11.86	
	4-methylcatechol	59.98	3.79		100.76	
	3-hydroxytyramine hydrochloride	10.36	9.79		6.74	
	Pyrogallol	29.93	19.21		9.93	
	Gallic acid	ND	ND		ND	
	3,4-dihydroxyphenylpropionic acid	4.57	101.21		0.29	
	Caffeic acid	120.73	16.64		46.23	
	Chlorogenic acid	42.21	6.30		42.67	
*Guankou* grape (*Vitis vinifera* × *Vitis labrusca*)	Catechol	2617.60	30.22	86.46		Wang et al. (2022)
	4-methylcatechol	5358.54	22.32	238.98		
	Catechinic acid	3557.76	4.89	727.39		
	Caffeic acid	1035.63	0.31	3,505.88		
**Vegetables**						
Globe artichoke (*Cynara scolymus*)^c^	Catechol	19662	10.2			Aydemir (2004)
	4-methylcatechol	12500	12.4			
	DL-3,4-dihydroxyphenylalanine	6060	36.3			
	L-3,4-dihydroxyphenylalanine Pyrogallol	5865	37.7			
		8065	14.3			
	Gallic acid	4620	43.6			
Yacon roots (*Smallanthus sonchifolius*)	Chlorogenic acid	1428.6	1.14	1253.2		Neves & da Silva (2007)
	4-methylcatechol	525.3	1.34	392.0		
	Caffeic acid	494.4	0.23	1890.4		
	Catechol	666.0	5.00	133.2		
Eggplant (*Solanum melongena*)^d^	4-methylcatechol	5.596	2.122	2.637		Todaro et al. (2011)
	Catechol	2.455	2.973	0.826		
	Chlorogenic acid	2.317	1.351	1.715		
	3,4-dihydroxyhydrocinnamic acid	5.452	2.122	2.569		
	Caffeic acid	2.826	6.149	0.46		
Purple Sweet Potato (Ipomoea batatas L. Lam)^d^	Catechol	200.00	0.62			Torres et al. (2021)
	Chlorogenic acid	270.18	0.22			
	Caffeic acid	140.84	0.22			
	4,5-dicaffeoylquinic acid	161.29	0.31			
	3,5- dicaffeoylquinic acid	277.78	0.14			
	3,4- dicaffeoylquinic acid	178.57	0.27			
	4,5-feruloyl-caffeoylquinic acid	212.77	0.68			
	3,4,5-tricaffeoylquinic acid	238.95	0.09			
	Peonidin 3-caffeoyl-p-hydroxybenzoyl sophoroside-5-glucoside	158.73	0.29			
**Seeds and Other Plant Material**						
Ferula sp. leaves^c^	Catechol	8541	2.34			Erat et al. (2006)
	4-methylcatechol	3502	6.58			
	Chlorogenic acid	5866	0.764			
	(+)-catechin	3372	0.554			
	(−)-epicatechin	6426	0.798			
Ferula sp. stems^c^	Catechol	4389	2.64			Erat et al. (2006)
	4-methylcatechol	2950	6.78			
	Chlorogenic acid	1764	1.07			
	(+)-catechin	4823	0.306			
	(−)-epicatechin	5308	2.89			
Lotus Seed (Nelumbo nucifera Gaertn.)	(−)-Epicatechin	1435.33	2.89			Li et al. (2021)
	Gallic acid	1247.67	4.46			
	(+)-catechin	1126.00	4.13			
	Chlorogenic acid	522.00	7.65			

U: Unit of enzyme activity defined as 0.001 increase in absorbance min^−1^ mL^−1^ from a UV-VIS spectrophotometric enzyme assay.

ND: not detected.

a *V*_max_ is reported as U mg^−1^ Protein x 10^3^. *V*_max_/*K*_*m*_ is reported as U mg^−1^ Protein mM^−1^ x 10^3^.

b *V*_max_ is reported as μM min^−1^.

c *K*_*m*_ is reported as moles L^−1^.

d The units for *V*_max_ and *K*_*m*_ are not reported.

For *o*-diphenol substrates, PPO is often reported to have high specificity for caffeic acid and chlorogenic acid (Li et al., 2023; Neves & da Silva, 2007; Wang et al., 2022; Wei et al., 2022). In contrast, PPO usually exhibits a low specificity for catechol (Derardja et al., 2017; Derardja et al., 2022; Neves & da Silva, 2007). Caffeic acid, chlorogenic acid and catechol all have an *o*-diphenol group but differ in the presence and composition of the attached side chain. While several theories exist to explain substrate specificity (McLarin and Leung, 2020), a recent study by Prexler et al. (2018) suggested differences in a particular amino acid residue can regulate specificity. PPOs that have a hydrophobic residue, such as isoleucine, adjacent to H_B2_, show preference for positively charged substrates; whereas if the residue is positively charged, such as arginine, the PPO exhibits higher specificity for substrates with negatively charged side chains, such as those present on caffeic acid and chlorogenic acid. Wei et al., 2021, Wei et al., 2022 reported that blueberry PPO has a hydrophobic leucine residue adjacent to H_B2_ and exhibits greater specificity for catechol than chlorogenic acid, in agreement with this mechanism. However, grape (*Vitis labrusca* x *Vitis vinifera*) PPO showed a preference for substrates with hydrophobic side chains, despite having a positively charge lysine residue adjacent to H_B2_ (Li et al., 2019). Substrate specificity is likely determined by a range of variables, rather than just one change in the amino acid sequence (McLarin and Leung, 2020). Given browning occurs in systems with multiple potential substrates, understanding what factors determine their relative oxidation rates is of key importance.

### 2.2 Polyphenol oxidase kinetics in complex systems

Foodstuffs are complex systems and competition between substrates may alter the enzyme kinetics *in vivo* (Kim et al., 2006). As described above, PPO catalyses the oxidation of *o*-diphenols to highly reactive *o*-quinones (Gawlik-Dziki et al., 2007). Flavonoids and caffeoylquinic acids are phenolic compounds that often have an *o*-diphenol functional group. Numerous studies have investigated the enzyme kinetics and specificity of PPO from different products (Table 1). However, this data is derived from simple reaction systems of purified enzyme and a single substrate, often using substrates not found naturally in the original food, such as catechol. While enzyme kinetics can shed light on the relative rates substrates are oxidised, it does not account for additional interactions between the generated *o*-quinones and substrates that occur in complex systems (Gawlik-Dziki et al., 2007).

Several groups have attempted to elucidate the specificity of PPO for competing substrates in complex systems. García-Rodríguez et al. (2011) investigated the influence of PPO on the oxidation of virgin olive oil (VOO) phenolics. An *in vitro* system comprising of extracted PPO and phenolics from VOO was incubated and the changes in individual phenolic concentrations monitored by high performance liquid chromatography-mass spectrometry (HPLC-MS). PPO was able to catalyse the oxidation of aglycon derivatives of oleuropein, but not the derivatives of ligstroside. This was attributed to olive PPO having catechol oxidase but not tyrosinase activity. The quantitative reduction in phenolics of interest was accompanied by the appearance of new chromatographic peaks. The authors proposed these new peaks were polymeric products of the oxidised phenolics. However, the identity of these polymeric products was not elucidated. This raises the question of what reactions are resulting in the formation of these polymeric products. Further work using this methodology requires a focus on identifying these new chromatographic peaks to confirm they are indeed derived from the initial phenolic compounds.

Wang et al. (2022) applied a more direct method to study the effect of PPO on phenolics during wine production using *Guankou* grapes (*Vitis vinifera* × *Vitis labrusca*). Taking aliquots directly from the wine fermentation vessel, seventeen different phenolic compounds were monitored over the 108 h fermentation, using Ultra High Pressure Liquid Chromatography (UHPLC), and all decreased in concentration (Wang et al., 2022). They also determined the PPO activity for several substrates, though caffeic acid and gallic acid were the only substrates tested that were present in the wine sample. Caffeic acid and catechin decreased as expected, as these are known substrates of PPO. However, PPO exhibited no activity towards gallic acid, and yet a decrease of more than 80% was observed during fermentation. Furthermore, other phenolics such as ferulic acid, resveratrol, p-coumaric acid also decreased. These phenolics are not substrates of PPO, as they lack an *o*-diphenol group. As with the decrease in phenolics observed by García-Rodríguez et al. (2011), the broad decreases observed here suggest other reactions taking place not directly catalysed by PPO. While this study identifies molecules that may be involved in enzymatic browning, further work is required to confirm their involvement, and which have the most significant impact on browning.

Untargeted metabolomics has the potential to simultaneously evaluate hundreds of molecules for association with enzymatic browning. Garcia et al. (2016) applied this technique to compare metabolites between two iceberg lettuce (*Lactuca sativa*) cultivars before and after five days of browning. While this method is not quantitative it is able to tentatively identify the direction of change for metabolites. Interestingly, Garcia et al. (2016) found chlorogenic acid, dicaffeoylquinic acid isomers, and dicaffeoyltartaric acid increased between day zero and day five, positively correlated with browning. These compounds are substrates for PPO and have been reported to decrease during enzymatic browning in other food products, such as purple sweet potato (Torres et al., 2021). A follow up trial by García et al. (2017) using two Romaine lettuce cultivars measured metabolite changes over five days but with additional time points. Chlorogenic acid and caffeoyltartaric acid both decreased substantially in the first 2 h, then chlorogenic acid began increasing and caffeoyltartaric acid resumed a slower decline. Mechanical wounding has been shown to up regulate the phenylpropanoid pathway to promote wound healing (Dixon et al., 2002; García et al., 2017). Chlorogenic acid is biosynthesised via this pathway, with its oxidation by PPO and subsequent transformation into brown pigments thought to be part of wound healing (García et al., 2017). It may be that the phenylpropanoid pathway is also up regulated in undamaged tissue; with a concomitant increase in chlorogenic acid (Saltveit, 2000). As PPO only interacts with phenolic compounds where cells are ruptured, chlorogenic acid oxidation would only be expected at the wounding site. This would also suggest PPO substrates are initially rapidly oxidised by PPO, with upregulation of their biosynthesis occurring later after wounding, leading to the eventual increase in concentration. However, how these concentration changes correlate with browning is unclear as the degree of browning was only measured on day zero and five. By using an untargeted approach, García et al. (2017) have been able to produce a comprehensive map of the metabolic changes, occurring during enzymatic browning, in lettuce. This approach allows for PPO substrates and non-substrates to be identified for subsequent targeted analyses.

## 3 Non-PPO substrates involved in enzymatic browning

Once *o*-diphenolic compounds are oxidised into *o*-quinones, they interact with other compounds to form brown pigmented melanins. The nature of these intermediary compounds is of interest, as they may regulate the rate and extent of browning. Several compound classes have been implicated such as anthocyanins, *o*-quinones, amino acids and phenolics that are not PPO substrates (Fang et al., 2007; Ito and Yanase, 2022; Liu et al., 2022; Mertens et al., 2019; Vissers et al., 2017). While the analysis of enzymatic browning and the associated intermediary compounds is complicated by the transient nature of intermediaries and difficulty in isolating high molecular weight melanin polymers; several groups have made progress in elucidating their nature (Mertens et al., 2021; Varga et al., 2016).

Anthocyanins are a polyphenolic class that exhibit red to blue colours observed in fresh produce (Herrera-Balandrano et al., 2021). Despite being polyphenolic compounds, many are not considered substrates for PPO, as they lack an *o*-diphenolic moiety (Qi et al., 2020). Cyanidin 3-glucoside (C3G) is the primary colourant in bayberry (*Myrica rubra* cv. Biqi) and has been shown to undergo degradation during enzymatic browning of blueberries (Kader et al., 1998). However, despite having an *o*-diphenolic group, it is not a substrate for PPO; this has been attributed to the glucoside group sterically hindering the docking of the compound with the PPO active site (Kader et al., 1998). Using an *in vitro* model system, Fang et al. (2007) proposed bayberry PPO could use gallic acid as a substrate with the resulting o-quinone undergoing oxidative coupling with C3G. However, the authors did not indicate whether the oxidative coupling with C3G resulted in brown pigments. However, Kader et al. (1998) reported that the initial degradation of C3G, in the presence of chlorogenic acid and PPO from blueberries, resulted in a loss of pigment colour. This was followed by a secondary condensation reaction between C3G and chlorogenic acid resulting in brown pigments. These findings suggest that non-PPO substrates can be intrinsic to the progression of enzymatic browning once o-quinones have been generated by PPO activity.

Similar intermediate condensation reactions have been linked to the formation of brown pigments during enzymatic browning of black tea. Catechins are polyphenols that have an o-diphenol group, found in high concentrations in tea leaves. During fermentation, PPO oxidises catechin resulting in its condensation into various polymers such as theaflavins and thearubigins (Ito and Yanase, 2022). Theaflavins are thought to be intermediaries of thearubigins which are the most prevalent brown pigments in black tea (Ito and Yanase, 2022). Using untargeted UHPLC-QTOF-MS, Ito and Yanase (2022) investigated the changes to catechins and their condensation products during black tea processing. They observed that theaflavins only accounted for a small proportion of the catechins oxidised during fermentation. Several large unknown compounds were positively correlated with thearubigin variation (*r* ≥ 0.4) and negatively correlated with total catechin content (*r* ≤ − 0.7). Fragment ions from these compounds suggested they were derived from catechins. The authors proposed that the theaflavins may not be the only intermediaries of the thearubigins as previously proposed, with other, as yet uncharacterised, compounds acting as intermediates. This highlights the possibility that non-PPO substrates, could be involved in the formation of thearubigins. An example of this is, the non-PPO substrate, epigallocatechin in theaflavin formation. PPO generated catechin o-quinones participate in redox reaction with epigallocatechin, allowing for subsequent polymerisation (Tanaka and Kouno, 2003). This sets precedent and alludes to a likely mechanism for non-PPO substrate involvement.

Reactive *o*-quinones can form adducts with the nucleophilic side chains of amino acids and proteins, eventually polymerising into high molecular weight brown pigments (Quan et al., 2019; Rawel and Rohn, 2010). However, some amino acids can act as anti-browning agents, depending on concentration and side chain nucleophilicity. Ali et al. (2016) investigated the impact of submersing cut potato tubers in different amino acid solutions on the extent of enzymatic browning. High concentrations of valine, methionine, phenylalanine, and glycine (1000 mM) all resulted in increases in browning, whereas low concentrations (< 100 mM) generally resulted in a reduction of browning. Cysteine resulted in a reduction in browning at all concentrations above 0.01 mM. Using a model PPO activity assay with catechol as the substrate, the authors proposed the formation of catechol-amino acid adducts as the intermediary for brown polymer formation. Specifically, they proposed the presence of glycine resulted in the condensation of the primary amine group to the 4’ and 5’ carbons of catechol to form diglycine-catechol products. While catechol is often used in assays to represent all *o*-diphenol containing molecules, it is worth noting that it is not a phenolic reported to be present in potato tubers (Sampaio et al., 2021). Moreover, phenolics which have been reported in potato tubers, such as chlorogenic acid and caffeic acid, already have a moiety associated with the dihydroxybenzene ring (Furrer et al., 2017). As such, the proposed diglycine-catechol product is not likely to be encountered *in vivo* during enzymatic browning of potato tubers. *In vitro* studies are essential in elucidating the non-enzymatic reactions products leading to browning, but care must be taken to ensure experimental results can be related back to *in vivo* conditions.

**Fig. 2:**
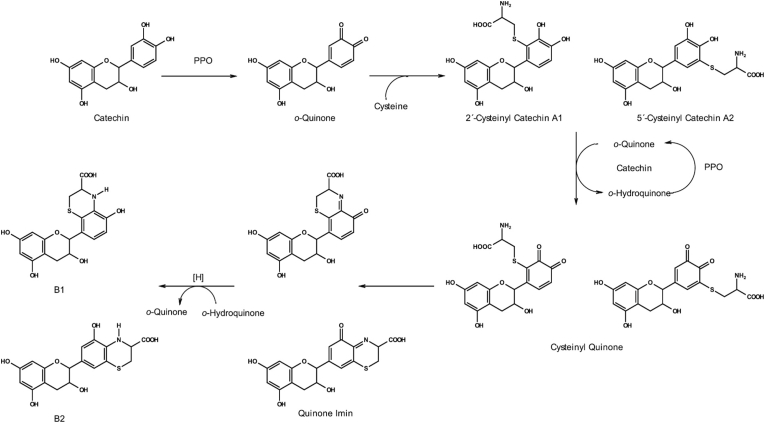
Proposed pathway for formation of dihydrobenzothiazine derivatives.

Source: (Mertens et al., 2019)

**Fig. 3:**
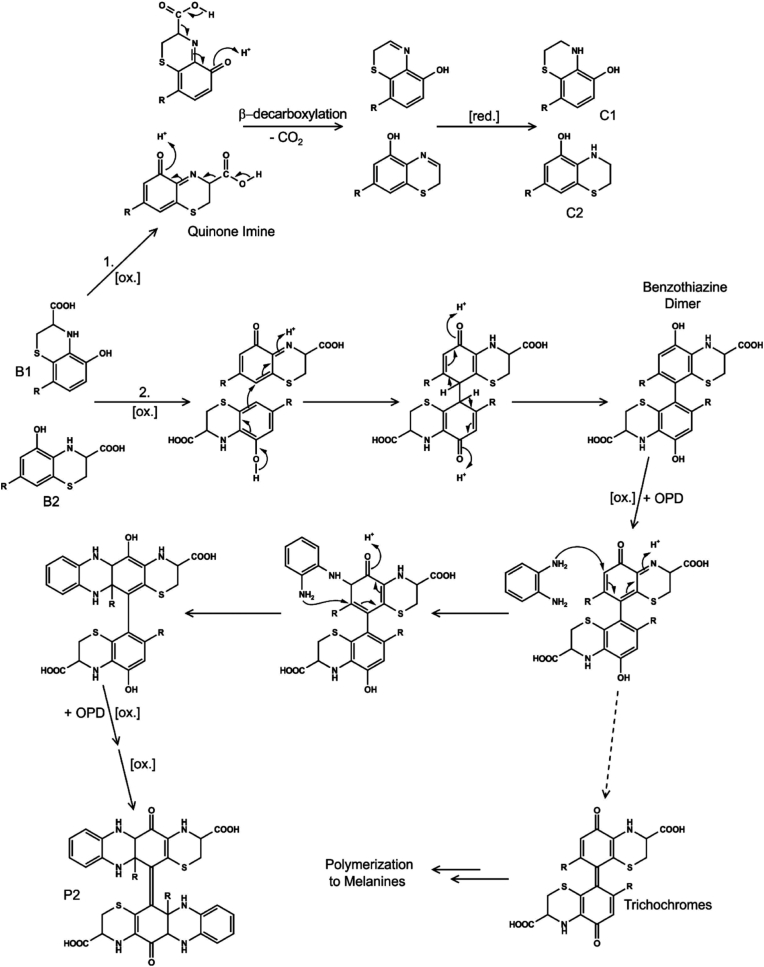
Proposed pathway for melanin formation from dihydrobenzothiazine derivatives.

Source: (Mertens et al., 2021)

In contrast to glycine, cysteine is generally accepted to be an inhibitor of enzymatic browning, but Mertens et al. (2019) demonstrated that certain cysteine adducts may undergo further transformation. Incubations of PPO, catechin, and cysteine were found to form catechin-cysteine adducts with subsequent intramolecular ring closure of the cysteine amino group producing the intermediary dihydrobenzothiazine derivatives (DHBT) (Fig. 2.). The formation of DHBT derivatives coincided with the appearance of unresolved complex mixtures in the chromatogram and the development of colour in the samples. The authors proposed that the unresolved mixture contained coloured melanin compounds. Further work to investigate the constituents of this mixture demonstrated that the DHBT intermediaries can undergo oxidative coupling to form dimers (Fig. 3.). These dimers are proposed to polymerise further into coloured melanin products (Heinrich et al., 2012; Mertens et al., 2021). The proposed formation of DHBT, improves on the model proposed by Ali et al. (2016), by taking into account the presence of the moiety's typically associated with the dihydroxybenzene ring of phenolics. Moreover, it provides strong evidence for the role of compounds, which are not PPO substrates, in the formation of brown pigments.

**Fig. 4:**
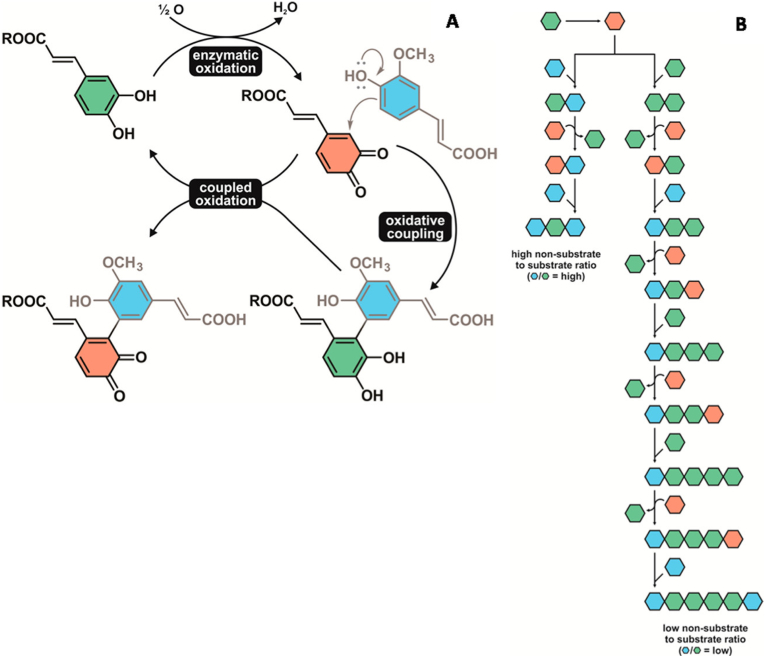
(A) Proposed model of oxidative coupling and redox cycling for caffeic acid esters and ferulic acid. (B) Impact of non-substrate to substrate ratio on progression of oxidative coupling.

Source: (Vissers et al., 2017)

In addition to amino acids such as cysteine, phenolics that are not PPO substrates are likely involved in enzymatic browning. Using a novel approach, Vissers et al. (2017) investigated the impact of specific phenolic compounds on the extent of enzymatic browning in sugar beet leaves (*Beta vulgaris*). Pools of fractionated phenolics from sugar beet leaves were incubated with extracted PPO and changes in phenolic concentrations were monitored. Caffeic acid esters, such as chlorogenic acid, were the primary substrate for PPO, as is widely accepted (Derardja et al., 2022; Torres et al., 2021). However, phenolics that did not possess an *o*-diphenol group also decreased in concentration. *In vitro* model systems with various quantities of PPO substrate and non-PPO substrate suggested that oxidative coupling of these compounds occurred as part of enzymatic browning. The authors proposed a model similar to that of Mertens et al. (2021) wherein, an *o*-quinone generated by PPO can either undergo oxidative coupling with a reduced *o*-diphenol or another nucleophilic compound, such as ferulic acid or sinapic acid. Additional units would couple to the dimer through redox cycling and subsequent oxidative coupling (Fig. 4.). Further, the authors proposed that the ratio of non-substrates to PPO substrates played a critical role in determining the extent of polymerisation and thus enzymatic browning. High ratios (1:8) would limit the potential for extension of the polymer and thus the development of brown pigments; whereas a low ratio (1:3, 1:1) would allow extensive polymerisation and brown pigment formation. The importance of redox cycling to the extension of phenolic polymers has been demonstrated more recently in apple juice (Liu et al., 2022) and wine (Ma and Waterhouse, 2018). The involvement of non-PPO substrates in the enzymatic browning process presents a new target for mitigating browning. Not only is the presence of these compounds relevant to enzymatic browning, but also their relative proportion compared to PPO substrates. The involvement of ferulic acid and sinapic acid has been demonstrated (Vissers et al., 2017), but other food products do not necessarily contain these compounds, raising the question of what other compounds may be involved.

## 4 A generalised mechanism for enzymatic browning

**Fig. 5:**
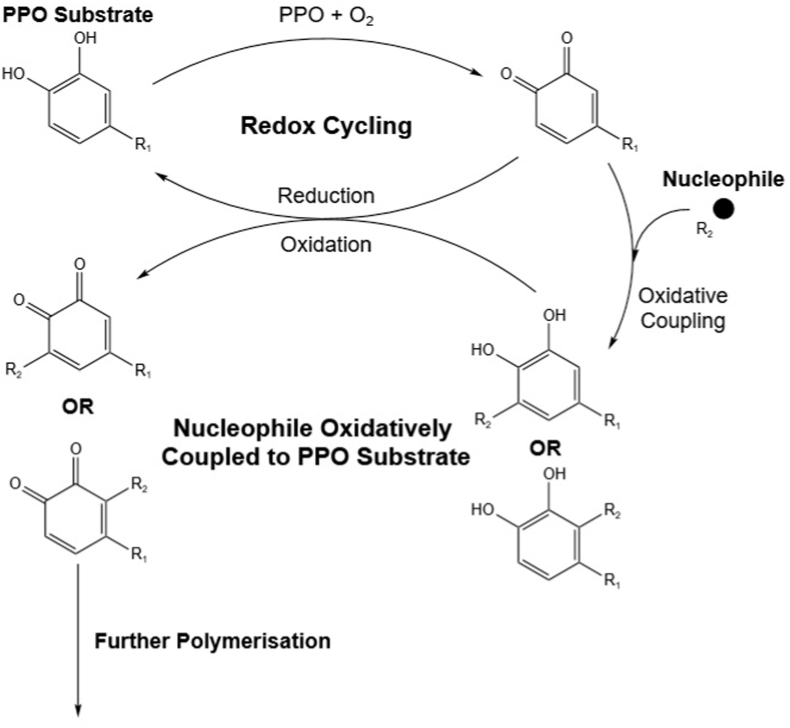
Generalised schematic for the oxidative coupling-redox cycling mechanism. R1: Phenolic structure; R2: nucleophilic molecule that then becomes an adduct at either the C-2’ or C-5’ position.

Several compound classes are viable candidates for non-enzymatic reactions with PPO products. However, this has only been demonstrated experimentally with a few molecules. A recurring theme reported by these authors is the involvement of redox cycling to facilitate oxidative coupling and subsequent polymerisation. This has been reported for amino acids and other non-PPO substrates such as ferulic acid and sinapic acid (Ali et al., 2016; Liu et al., 2022; Ma and Waterhouse, 2018; Mertens et al., 2019, 2021; Vissers et al., 2017). The mechanism described by these authors can be generalised schematically (Fig. 5). The *o*-diphenol group of a phenolic compound is oxidised by PPO resulting in an *o*-quinone, which can subsequently proceed through two reactions. One reaction is to undergo oxidative coupling with a nucleophile, such as another phenolic compound, amino acid or other non-PPO substrate (Ali et al., 2016; Ma and Waterhouse, 2018; Mertens et al., 2019; Vissers et al., 2017). This results in a dimer where the *o*-quinone group is reduced back to an *o*-diphenol, which can be oxidised again. The second reaction pathway is for the *o*-quinone to be reduced as part of a redox cycle. As the *o*-quinone is reduced it can oxidise a dimer, making it available for further cycles of oxidative coupling. By this cyclical reaction mechanism polymerisation of the initial phenolic compound can proceed, leading to large brown pigmented polymers (Mertens et al., 2021). This generalised model provides a mechanistic foundation for further investigation into the involvement of other non-PPO substrates in the formation of brown pigments. Understanding what molecules are involved in the non-enzymatic reactions leading to browning may facilitate the development of new novel interventions for its mitigation.

## 5 Prospective analytical approaches

Enzymatic browning research has traditionally focussed characterising polyphenol oxidase structurally, biochemically, and kinetically. As such, many of the current mitigation practices are focussed on inhibition of the enzyme. However, these practices can have negative side effects on the products sensory or nutritional quality. Recent advances in understanding enzymatic browning, highlight the importance of the subsequent non-enzymatic reactions and the molecules involved. Indeed, PPO activity does not directly generate brown pigments, it is the subsequent reactions that are the critical step. Comprehensive workflows and utilising a broader range of analytical techniques are needed to progress knowledge of these reactions; and subsequently identify new mitigation techniques for browning.

Enzymatic browning involves a large number of substrates *in vivo*. García et al. (2017) demonstrated that untargeted UPLC-ESI-QTOF-MS can provide a comprehensive qualitative screening of molecules during browning. This data can then be evaluated for correlations with the development of browning. Collection of time series data can provide even greater insight into the molecular changes occurring as browning progresses. Identification of potential molecular candidates involved in browning can then be followed up with subsequent analysis.

*In vitro* assays can complement untargeted analyses by providing mechanistic insight into non-enzymatic reactions occurring during browning. Recently, Yu et al. (2023) and Liu et al. (2022) demonstrated this by conducting *in vitro* assays of different *o*-quinones and non-PPO substrate combinations to elucidate the reaction kinetics between them. Both studies utilised HPLC-MS platforms to quantify the changes in concentration of the substrates. ^13^C isotope labelling of substrates is a recent advancement in this area, allowing substrates to be traced through the complex reaction pathways (Geng et al., 2023). Furthermore, this approach could allow for the identification of the polymerisation products, which has not been achieved with current analytical techniques.

Non-enzymatic reactions occurring during browning are primarily redox reactions. Nucleophiles participating in redox reactions each have a distinct oxidation and reducing potential. These potentials can define if and at what rate they interact with oxidising agents, such as *o*-quinones. Cyclic voltammetry is a well-established electrochemical technique for quantifying the potential of molecules (Elgrishi et al., 2018). Quantifying the oxidation potential of nucleophiles and the reduction potential of *o*-quinones can indicate the likelihood of the reaction occurring. Furthermore, cyclic voltammetry be used to gain mechanistic insight into the fate of the reactants (Elgrishi et al., 2018). Cyclic voltammetry has been used to characterise the antioxidant capacity of wines (Kilmartin et al., 2001; Oliveira et al., 2016). However, it has not been widely used to study enzymatic browning.

While these assays can provide mechanistic insight into non-enzymatic reactions, complimentary evidence of their role in browning is required. Many studies utilise the absorbance at 420 nm of a liquid sample to demonstrate the progression of browning (Ma and Waterhouse, 2018; Mertens et al., 2021; Plazas et al., 2013; Yu et al., 2023). However, this only captures a fraction of the visible spectrum that is absorbed when browning is observed. An alternative approach is the use of a colourimeter and the CIEL*a*b* colour space. This system represents colour using three coordinate values, derived from reflectance over the whole visible light spectrum (Pérez-Magariño and González-Sanjosé, 2003). When monitoring the browning of globe artichokes (*Cynara scolymus*), Cabezas-Serrano et al. (2009) observed the a* value increased substantially, indicating an increase in red hue. Measurement at 420 nm alone, may not have captured the full magnitude of colour change. Where CIEL*a*b* system cannot be used, absorbance across the full visible spectrum should be evaluated as Cui et al. (2023) recently demonstrated.

Elucidating the non-enzymatic reactions in browning requires the combining of multiple analytical and experimental techniques. High resolution untargeted mass spectrometry analytics will be essential in identifying potential non-PPO substrate interactions during enzymatic browning. Likewise, targeted mass spectrometry techniques will also be essential for elucidating the reaction mechanisms from *in vitro* experiments. The combining of these powerful platforms with complimentary voltammetric and spectrophotometric methods can provide the mechanistic understanding needed to identify future anti-browning solutions.

## 6 Conclusion

Enzymatic browning is a pervasive issue for the food industry. The oxidation of polyphenols by PPO is the first step in a complex system of reactions that results in brown pigmented melanin compounds. The prevailing theory has been that enzymatic browning can be satisfactorily characterised by enzyme kinetics and substrate specificity. However, it is becoming clear that the involvement of non-PPO substrates is of significance. A common mechanism of oxidative coupling facilitated by redox cycling can be seen as driving the development of high molecular weight melanin polymers. The models proposed by Ito and Yanase (2022), Mertens et al. (2021), and Vissers et al. (2017) all share this same underlying mechanism. However, while our mechanistic understanding of enzymatic browning has expanded, the possible involvement of, as yet unidentified, non-PPO substrates warrants further investigation. Further research is needed to identify which substrates or non-substrates contribute most to the development of enzymatic browning; potentially allowing for new targeted preventative action.

## Funding

This research is supported by the Future Food Systems Co-operative Research Centre; the first author is supported by an 10.13039/100015539Australian Government Research Training Program scholarship.

## CRediT authorship contribution statement

**Andrew Tilley:** Conceptualization, Investigation, Writing – original draft. **Mark P. McHenry:** Conceptualization, Supervision, Writing – review & editing, Funding acquisition. **Julia Anwar McHenry:** Supervision, Writing – review & editing, Funding acquisition. **Vicky Solah:** Conceptualization, Supervision, Writing – review & editing. **Kirsty Bayliss:** Supervision, Writing – review & editing.

## Declaration of competing interest

The authors declare that they have no known competing financial interests or personal relationships that could have appeared to influence the work reported in this paper.

## Data Availability

No data was used for the research described in the article.
